# Supplementary material for: Genetic structure and diversity of the selfing model grass Brachypodium stacei (Poaceae) in Western Mediterranean: out of the Iberian Peninsula and into the islands
Source: PeerJ. 2016 Sep 8;4:e2407. doi: 10.7717/peerj.2407 (PMC5018678; doi:10.7717/peerj.2407)
Supplement: Supplemental Information 1 [file peerj-04-2407-s001.pdf]

Table S1. List of alelles genotyped in Brachypodium stacei.

Title: Brachypodium stacei
LOCUS 1
LOCUS 2
LOCUS 3
LOCUS 4
LOCUS 5
LOCUS 6
LOCUS 7
LOCUS 8
LOCUS 9
LOCUS 10
Pop
GRA , 342342 340340 166166 203203 180180 184184 294294 157157 238238 250250
GRA , 342342 340340 166166 203203 180180 184184 294294 145145 238238 250250
GRA , 342342 340340 166166 203203 180180 184184 294294 157157 238238 250250
GRA , 342342 340340 166166 203203 180180 184184 294294 157157 238238 250250
GRA , 342342 340340 166166 203203 180180 184184 294294 157157 238238 250250
GRA , 342342 340340 166166 187203 180180 184184 294294 157157 238238 250250
GRA , 342342 340340 166166 203203 180180 184184 294294 157157 238238 250250
GRA , 342342 340340 166166 203203 180180 184184 294294 157157 238238 250250
GRA , 342342 340340 166166 203203 180180 184184 294294 157157 238238 250250
GRA , 342342 340340 166166 203203 180180 184184 294294 157157 238238 250250
Pop
ALM , 342342 340340 180180 203203 182182 184184 294294 157157 236236 250250
ALM , 342342 340340 180180 203203 182182 184184 294294 157157 236236 250250
ALM , 342342 340340 180180 203203 182182 184184 294294 157157 236236 250250
ALM , 342342 340340 180180 203203 182182 184184 294294 157157 236236 250250
ALM , 342342 340340 180180 203203 182182 184184 294294 157157 236236 250250
ALM , 342342 340340 180180 203203 182182 184184 294294 157157 236236 250250
ALM , 342342 340340 180180 203203 182182 184184 294294 157157 236236 250250
ALM , 318342 340340 180180 203203 182182 184184 294294 157157 236236 250250
ALM , 342342 340340 180180 203203 182182 184184 294294 157157 236236 250250
ALM , 342342 340340 180180 203203 182182 184184 294294 157157 236236 250250
Pop
JAE1 , 342342 340340 180180 205205 182182 184184 294294 157157 236236 250250
JAE1 , 342342 340340 180180 205205 182182 184184 294294 157157 236236 250250
JAE1 , 342342 340340 180180 205205 182182 184184 294294 157157 236236 250250
JAE1 , 342342 340340 166180 205205 182182 184184 294294 157157 236236 250250
JAE1 , 342342 340340 180180 205205 182182 184184 294294 157157 236236 250250
JAE1 , 342342 340340 180180 205205 182182 180180 294294 157157 236236 250250
JAE1 , 342342 340340 166180 205205 182182 180180 294294 157157 236236 250250
JAE1 , 342342 340340 180180 205205 182182 180180 294294 157157 236236 250250
JAE1 , 342342 340340 180180 205205 182182 180180 294294 157157 236236 250250
JAE1 , 342342 340340 180180 205205 182182 184184 294294 157157 236236 250250
Pop
JAE2 , 342342 340340 180180 203203 182182 184184 294294 157157 236236 250250
JAE2 , 342342 340340 180180 203203 182182 184184 294294 157157 236236 250250
JAE2 , 342342 340340 180180 203203 182182 184184 294294 157157 236236 250250
JAE2 , 342342 340340 180180 203203 182182 184184 294294 157157 236236 250250
JAE2 , 342342 340340 180180 203203 182182 184184 294294 157157 236236 250250
JAE2 , 342342 340340 180180 203203 182182 184184 294294 157157 236236 250250
JAE2 , 342342 340340 180180 203203 182182 184184 294294 157157 236236 250250
JAE2 , 342342 340340 180180 203203 182182 184184 294294 157157 236236 250250
JAE2 , 342342 340340 180180 203203 182182 184184 294294 157157 236236 250250
JAE2 , 342342 340340 180180 203203 182182 184184 294294 157157 236236 250250

Pop
PORT , 342342 340340 180180 203203 176176 194194 294294 157157 240240 250250
PORT , 342342 340340 180180 203203 176176 194194 294294 157157 240240 250250
PORT , 342342 340340 180180 203203 176176 194194 294294 157157 240240 250250
PORT , 342342 340340 180180 203203 176176 194194 294294 157157 240240 250250
PORT , 342342 340340 180180 203203 176176 194194 294294 157157 240240 250250
PORT , 342342 340340 180180 203203 176176 194194 294294 157157 240240 250250
PORT , 342342 340340 180180 203203 176176 194194 294294 157157 240240 250250
PORT , 342342 340340 180180 203203 176176 194194 294294 157157 240240 250250
PORT , 342342 340340 180180 203203 176176 194194 294294 157157 240240 250250
PORT , 342342 340340 180180 203203 176176 194194 294294 157157 240240 250250
Pop
CALBN , 342342 340340 180180 203203 176176 194194 294294 157157 238238 250250
CALBN , 342342 340340 180180 203203 176176 194194 294294 157157 238238 250250
CALBN , 342342 340340 166180 203203 176176 194194 294294 157157 238238 250250
CALBN , 342342 340340 180180 203203 176176 194194 294294 157157 238238 250250
CALBN , 318342 340340 180180 203203 176176 194194 294294 157157 238238 244250
CALBN , 342342 340340 184184 203203 176176 194194 294294 157157 238238 250250
CALBN , 342342 340340 184184 203203 176176 194194 294294 157157 238238 250250
CALBN , 342342 340340 180180 203203 176176 194194 294294 157157 238238 250250
CALBN , 342342 340340 180180 203203 176176 194194 294294 157157 238238 250250
CALBN , 342342 340340 180180 203203 176176 194194 294294 157157 238238 250250
Pop
CALBA , 342342 340340 180180 203203 176176 182182 294294 157157 238238 250250
CALBA , 342342 340340 180180 203203 176176 182182 294294 157157 238238 250250
CALBA , 342342 340340 180180 203203 176176 182182 294294 157157 238238 250250
CALBA , 342342 340340 180180 203203 176176 182182 294294 157157 238238 250250
CALBA , 342342 340340 184184 203203 176176 182182 294294 157157 238238 250250
CALBA , 342342 340340 184184 203203 176176 180196 294294 157157 238238 250250
CALBA , 342342 340340 180184 203203 176176 198198 294294 157181 238238 250250
CALBA , 342342 340340 184184 203203 176176 182198 294294 157157 238238 250250
CALBA , 342342 340340 180180 203203 176176 182182 294294 157157 238238 250250
CALBA , 342342 340340 180180 203203 176176 182182 294294 157157 238238 250250
Pop
CALREL , 342342 340340 180180 203203 176176 182182 294294 157157 238238 250250
CALREL , 318342 340340 180180 203203 176176 182182 294294 157157 238238 250250
CALREL , 342342 340340 180180 203203 176176 182182 294294 157157 238238 250250
CALREL , 342342 340340 180180 203203 176176 182182 294294 157157 238238 250250
CALREL , 342342 340340 184184 203203 176176 182182 294294 157181 238238 250250
CALREL , 342342 340340 184184 203203 176176 182182 294294 157157 238238 250250
CALREL , 342342 340340 180180 203203 176176 182182 294294 157181 238238 250250
CALREL , 342342 340340 184184 203203 176176 182182 294294 157157 238238 250250
CALREL , 342342 340340 180180 203203 176176 182182 294294 157181 238238 250250
CALREL , 342342 340340 180180 203203 176176 182182 294294 157157 238238 250250
Pop
ALI , 342342 340340 180180 203203 176176 182182 294294 157157 238238 250250
ALI , 342342 340340 180180 203203 176176 182182 294294 157157 238238 250250
ALI , 342342 340340 180180 203203 176176 182182 294294 157157 238238 250250
ALI , 342342 340340 180180 203203 176176 182182 294294 157157 238238 250250
ALI , 342342 340340 180180 203203 176176 182182 294294 157157 238238 250250
Pop
MEN , 342342 340340 180180 203203 190190 184198 294294 157157 232232 242250
MEN , 342342 340340 180180 203203 190190 184198 294294 145145 232232 250250
MEN , 342342 340340 180180 203203 190190 198198 294294 157157 232232 250250
MEN , 342342 340340 180180 203203 190190 198198 294294 157157 232232 250250
MEN , 342342 340340 180180 203203 190190 198198 294294 157157 232232 250250
MEN , 342342 340340 180180 203203 190190 198198 294294 157157 232232 250250

MEN , 342342 340340 180180 203203 190190 198198 294294 157157 232232 250250
MEN , 342342 340340 180180 203203 190190 198198 294294 157157 232232 250250
MEN , 342342 340340 180180 203203 190190 198198 294294 157157 232232 250250
MEN , 342342 340340 180180 203203 190190 198198 294294 157157 232232 250250
Pop
DRAG , 342342 340340 180180 203203 190190 184184 294294 157157 238238 250250
DRAG , 342342 340340 180180 189205 182182 180180 294294 157157 238238 250250
DRAG , 342342 340340 180180 203203 182182 180180 294294 157157 238238 250250
DRAG , 342342 340340 180180 203203 182182 180180 294294 157157 238238 250250
DRAG , 342342 340340 180180 203203 182182 180180 294294 157157 238238 250250
DRAG , 342342 340340 180180 203203 182182 180180 294294 157157 238238 250250
DRAG , 342342 340340 180180 203203 188188 180180 294294 157157 238238 250250
DRAG , 342342 340340 180180 203203 188188 180180 294294 157157 238238 250250
DRAG , 342342 340340 180180 203203 182182 180180 294294 157157 238238 250250
DRAG , 342342 340340 184184 203203 188188 180180 294294 157157 238238 250250
Pop
ARTA , 342342 340340 180180 203203 182182 184184 294294 157157 236236 250250
ARTA , 342342 340340 180180 203203 182182 184184 294294 157157 238238 250250
ARTA , 342342 340340 180180 203203 182182 184184 294294 157157 238238 250250
ARTA , 342342 340340 180180 203203 182182 184184 294294 157157 238238 250250
ARTA , 342342 340340 180180 203203 182182 184184 294294 157157 238238 250250
ARTA , 342342 340340 180180 203203 182182 184184 294294 157157 238238 250250
ARTA , 342342 340340 180180 203203 182182 184184 294294 157157 238238 250250
ARTA , 342342 340340 168180 203203 182182 184184 294294 157157 238238 250250
ARTA , 342342 340340 180180 203203 182182 184184 294294 157157 238238 250250
ARTA , 342342 340340 180180 203203 182182 184184 294294 157157 238238 250250
Pop
CAMPA , 342342 340340 180180 189205 190190 184184 294294 157157 238238 250250
CAMPA , 342342 340340 180180 203203 182182 184184 294294 157157 238238 250250
CAMPA , 342342 340340 180180 203203 182182 184184 294294 157157 238238 250250
CAMPA , 342342 340340 180180 203203 182182 184184 294294 157157 238238 250250
CAMPA , 342342 340340 180180 203203 182182 184184 294294 157157 238238 250250
CAMPA , 342342 340340 184180 189205 190190 184184 294294 157157 238238 250250
CAMPA , 342342 340340 180180 203203 182190 184184 294294 157157 238238 250250
CAMPA , 342342 340340 180180 203203 182182 184184 294294 157157 238238 250250
CAMPA , 342342 340340 180184 203203 182190 184184 294294 157157 238238 250250
CAMPA , 342342 340340 184184 203203 182182 184184 294294 157157 238238 250250
Pop
ALCU , 342342 340340 180180 203203 182182 184184 294294 157157 238238 250250
ALCU , 342342 340340 180180 203203 182182 184184 294294 157157 238238 250250
ALCU , 342342 340340 180180 203203 182182 184184 294294 157157 238238 250250
ALCU , 342342 340340 180180 203203 182182 184184 294294 157157 238238 250250
ALCU , 342342 340340 180180 203203 182182 184184 294294 157157 238238 250250
ALCU , 342342 340362 168180 203203 176182 184198 294294 157157 238238 250250
ALCU , 342342 340340 180180 203203 182182 184184 294294 157157 238238 250250
ALCU , 342342 340340 180180 203203 182182 184184 294294 157157 238238 250250
ALCU , 342342 340340 180180 203203 182182 184184 294294 157157 238238 250250
ALCU , 342342 340340 180180 203203 182182 184184 294294 157157 238238 250250
Pop
FELEN , 342342 340340 180180 203203 182190 180180 294294 157157 238238 250250
FELEN , 342342 340340 180180 203203 182182 180180 294294 157157 238238 250250
FELEN , 342342 340340 180180 203203 182182 180180 294294 157157 238238 250250
FELEN , 342342 340340 180180 203203 182182 180180 294294 157157 238238 250250
FELEN , 342342 340340 180180 203203 182182 180180 294294 157157 238238 250250
FELEN , 342342 340340 180180 189205 182182 180180 294294 157157 238238 250250
FELEN , 342342 340340 180180 203203 182182 180180 294294 157157 238238 250250
FELEN , 342342 340340 180180 203203 182182 180180 294294 157157 238238 250250

FELEN , 342342 340340 180180 203203 182182 180180 294294 157157 220238 250250
FELEN , 342342 340340 180180 203203 182182 180180 294294 157157 238238 250250
Pop
BONA , 342342 340340 180180 203203 182182 180180 294294 157157 238238 250250
BONA , 342342 340340 180180 205205 180180 180180 294294 157157 238238 250250
BONA , 342342 362362 166180 203203 174188 198198 294294 157181 220238 242250
BONA , 342342 362362 180180 205205 188188 184198 294294 157157 220238 242250
BONA , 342342 362362 180180 187203 174188 198198 294294 157157 220238 242250
BONA , 342342 340340 180180 203203 174188 198198 294294 157157 238238 250250
BONA , 342342 340340 180180 205205 174188 180180 294294 157157 238238 250250
BONA , 342342 362362 180180 203203 174188 198198 294294 157157 220238 250250
BONA , 342342 362362 180180 203203 174188 198198 294294 157187 220238 250250
BONA , 342342 340340 180180 205205 174188 180180 294294 157157 220238 250250
Pop
BANYA , 342342 340340 180180 203203 190190 198198 294294 145157 240240 250250
BANYA , 342342 340340 180180 203203 182182 180180 294294 145157 240240 250250
BANYA , 342342 340340 180180 203203 182182 180180 294294 157157 240240 250250
BANYA , 342342 340340 180180 189189 182182 180180 294294 157157 240240 250250
BANYA , 342342 340340 180180 189189 182182 184184 294294 157157 240240 250250
BANYA , 342342 340340 180180 203203 182182 184184 294294 157157 240240 250250
Pop
GOM , 342342 340340 180180 203203 180180 180180 294294 157157 220220 250250
GOM , 342342 340340 180180 203203 180180 180180 294294 157157 238238 250250
GOM , 342342 340340 180180 203203 180180 180180 294294 157157 238238 250250
GOM , 342342 340340 180180 203203 180180 180180 294294 157157 238238 250250
GOM , 342342 340340 180180 203203 180180 180180 294294 157157 238238 250250
GOM , 342342 340340 180180 203203 180180 180180 294294 157157 238238 250250
GOM , 342342 340340 180180 203203 180180 180180 294294 157157 238238 250250
GOM , 342342 340340 180180 203203 180180 180180 294294 157157 238238 250250
GOM , 342342 340340 180180 203203 180180 180180 294294 157157 238238 250250
GOM , 342342 340340 180180 203203 180180 180180 294294 157157 238238 250250
Pop
LAN , 342342 340340 180180 203203 180180 184184 294294 157157 238238 250250
LAN , 342342 340340 180180 203203 180180 184184 294294 157157 238238 250250
LAN , 342342 340340 180180 203203 180180 184184 294294 157157 238238 250250
LAN , 342342 340340 180180 205205 180180 184184 294294 157157 238238 250250
LAN , 342342 340340 180180 203203 180180 184184 294294 157157 238238 250250
LAN , 342342 340340 180180 203203 180180 184184 294294 157157 238238 250250
LAN , 342342 340340 180180 203203 180180 184184 294294 157157 238238 250250
LAN , 342342 340340 180180 205205 180180 184184 294294 157157 238238 250250
LAN , 342342 340340 180180 203203 180180 184184 294294 157157 238238 250250
LAN , 342342 340340 180180 203203 180180 184184 294294 157157 238238 250250
